# Human Cytomegalovirus Seroprevalence Among Blood Donors in the Madinah Region, Saudi Arabia

**DOI:** 10.7759/cureus.21860

**Published:** 2022-02-03

**Authors:** Waleed Mahallawi, Omar F Khabour, Abdullah Al-Saedi, Ziyad Almuzaini, Nadir Ibrahim

**Affiliations:** 1 Medical Laboratory Technology Department, College of Applied Medical Sciences, Taibah University, Madinah, SAU; 2 Department of Medical Laboratory Sciences, Faculty of Applied Medical Sciences, Jordan University of Science and Technology, Irbid, JOR

**Keywords:** immunocompromised, madinah, seroprevalence, blood donors, hcmv

## Abstract

Background and objective

Human cytomegalovirus (HCMV), a double-stranded DNA virus of the *Herpesviridae* family, can remain latent for long periods of time. HCMV may cause severe illness in immunocompromised patients and is associated with congenital anomalies. This study aimed to determine the anti-HCMV immunoglobulin G (IgG) and IgM antibody seroprevalence among blood-donating Saudi men in the Madinah region.

Methods

A total of 375 blood-donating Saudi men were recruited from the Central Blood Bank in Madinah, the Kingdom of Saudi Arabia (KSA), and stratified into three age groups: 18-30, 31-40, and 41-61 years. Anti-HCMV IgG and IgM antibody levels were measured by enzyme-linked immunosorbent assay (ELISA). Pearson’s correlation coefficient was used to correlate antibody levels with variables.

Results

Most of the tested samples (95.73%, n=356) were positive for anti-HCMV IgG antibodies, but only 1.6% (n=6) were positive for both IgM and IgG antibodies, and all of them belonged to the age groups of 31-40 and 41-61 years. A strong inverse correlation was found between anti-HCMV IgG antibody levels and age (r=−0.51, p<0.0001). Additionally, there was an inverse correlation between anti-HCMV IgG antibody levels and body mass index (BMI) (r=−0.11, *p*=0.036). No correlations were found between anti-HCMV IgG levels and hemoglobin levels or blood groups of the participants.

Conclusions

Blood-donating Saudi men in Madinah had a high seroprevalence of anti-HCMV IgG antibodies, indicating previous viral exposure. Age and BMI might influence the humoral immunologic memory response against HCMV, which appears to be endemic in Madinah.

## Introduction

Human cytomegalovirus (HCMV) belongs to the *Herpesviridae* family and is one of the most common herpes viruses to infect humans [1]. In most healthy individuals, HCMV infection is asymptomatic [2]. However, HCMV infection can cause severe illness and even death among immunocompromised patients, pregnant women, neonates, and transplant patients [3-5]. Congenital infections have been associated with developmental abnormalities, jaundice, and central nervous system diseases [6]. The reactivation of latent HCMV infections has been reported in some cases and is typically limited to certain cell types, particularly fully differentiated monocytes and macrophages. The ability of HCMV to remain latent and become reactivated is attributed to several immune evasion strategies [7]. The reactivation of HCMV can result in lethal disease presentations in acquired immunodeficiency syndrome (AIDS) patients and in immunocompromised patients who receive blood transfusions. Although the HCMV latency site is unknown, blood cells have been identified as a potential viral reservoir, which could cause reactivation [8].

During the primary infection period, infected persons shed the virus via saliva, breast milk, urine, sperm, and blood. Viral transmission can occur horizontally from person to person and vertically from mother to fetus through the placenta. In most individuals, the primary infection can be cleared without complications, and the immune system is able to preserve a good and effective balance to avoid CMV reactivation [9,10]. The risks associated with HCMV transmission through blood products have been demonstrated in several studies [11,12]. Blood transfusion is considered to be a significant source of HCMV infection, and transfusion-transmitted CMV (TT-CMV) in CMV-seronegative immunocompromised patients can result in fatal CMV disease [13]. However, blood banks do not routinely screen for HCMV in several countries, including the Kingdom of Saudi Arabia (KSA) [14,15].

The reported global HCMV seroprevalence varies widely among geographical regions, with a seroprevalence of 66% in the European region, 75% in South and North America, 86% in the Southeast Asian region, 88% in Africa and the Western Pacific, and 90% in the Eastern Mediterranean region [16]. Clinical indices for the HCMV infection include the destruction of myelopoiesis, hepatosplenomegaly, lymphadenopathy, thrombocytopenia, hemolytic anemia, and oral microsites. HCMV mononucleosis is characterized by prolonged fever, weight loss, and lymphocytosis in immunocompetent patients [17,18].

In the current study, we surveyed the seroprevalence of immunoglobulin G (IgG) and IgM anti-HCMV antibodies among male blood donors at the Central Blood Bank in Madinah, KSA. The study findings might assist in the prevention of HCMV transmission to at-risk transfusion recipients who suffer from immunopathology disorders.

## Materials and methods

Study design

This study used a cross-sectional observational design and was conducted from April to June 2021. The study was reviewed and approved by the Scientific Research Ethics Committee at the College of Applied Medical Sciences, Taibah University (IRB: 2021-2-MLT). Written informed consent was obtained from all subjects after they were fully informed about the study objectives and procedures. To ensure confidentiality, all samples were coded, and access to the data was limited to the research team.

Sample collection

Recruitment was conducted at the Central Blood Bank, Madinah, KSA. Approximately 819 healthy Saudi blood donors were invited to participate, of whom 375 men completed the study. A total of 5 ml of blood was collected from each subject in plain tubes. After the samples were coagulated, the serum was separated by centrifugation at 3000 × g for five minutes. Samples were stored at a temperature of −20 °C until they were assayed. All participants in the current study were men because very few female blood donors were identified during the study period; therefore, we did not recruit any female participants.

Inclusion and exclusion criteria

All samples included in the study were obtained from fit and eligible Saudi men (aged ≥18 years) who agreed to participate in the study. Donors of other nationalities were excluded from the study.

Enzyme-linked immunosorbent assay

All samples were tested for anti-HCMV IgM and IgG antibodies using enzyme-linked immunosorbent assay (ELISA). Cytomegalovirus IgG and IgM ELISA antibody assays were performed semi-automatically according to the manufacturer's instructions. All serum samples were diluted at 1:100 by adding 10 µl of serum and 1000 µl of dilution buffer and were tested in duplicate.

Measurement of anti-HCMV IgG antibodies

The anti-HCMV IgG antibody was measured using the commercial assay ENZYWELL Cytomegalovirus IgG reagent kit (DIESSE Diagnostica Senese, Monteriggioni, Siena, Italy). According to the manufacturer, the sensitivity of the test is 99.3% and the specificity is 99.8%.

In brief, sera and undiluted calibrators provided in the kits were incubated in microplates for 45 minutes at 37 °C. Then, wells were washed and incubated with the conjugate for 45 minutes at 37 °C. Tetramethylbenzidine (TMB) substrate was added after the second round of washing and incubated and protected from light for 15 minutes at room temperature. Afterward, the enzymatic reaction was stopped with a stop solution, and the absorbance was measured at 450 nm within 30 minutes. The average OD of the calibrators containing known concentrations of anti-HCMV IgG antibodies were plotted on a graph and the anti-HCMV IgG concentration of the test samples was obtained by extrapolation. Serum samples with anti-HCMV IgG concentrations of >12 EU/ml or 1.2 IU/ml were considered positive.

Measurement of anti-HCMV IgM antibodies

Anti-HCMV IgM antibody concentration was measured with commercial SERION ELISA classic CMV IgM (Institute Virion\Serion GmbH, Würzburg, Germany). According to the manufacturer, the test sensitivity is 97% and the specificity is 99%.

Sera from patients, controls, and standard sera were incubated in microplates for 60 minutes. Then, wells were washed and incubated with the antihuman IgM conjugate for 30 minutes. After the second round of washing, para-Nitrophenylphosphate (pNPP) substrate was added and incubated for 30 minutes. Then, the stop solution sodium hydroxide was added immediately into all wells and the absorbance was measured at 405 nm. Antibody activities in U/ml were determined by converting optical density signals from the lot-specific standard curve. Anti-HCMV IgM concentrations of ≥15 U/ml were considered positive. All washing and reading steps were run using a semi-automated ELISA washer and reader (Biotek, Winooski, VT) according to the user manual provided by the manufacturer.

Statistical analysis

GraphPad Prism 9.22 (GraphPad Software Inc., San Diego, CA) was used to analyze anti-HCMV IgG antibody levels in relation to age, body mass index (BMI), hemoglobin levels, and blood groups. Differences between anti-HCMV IgG antibody levels among age groups were assessed by comparing the median values using a two-tailed Mann-Whitney U test. The Pearson's correlation coefficient with a 95% confidence interval was used to evaluate relationships between variables. A p-value ≤0.05 was considered statistically significant.

## Results

Demographic data

A total of 375 subjects were included in this study. The mean age of the participants was 32.72 ± 10.82 years. The mean BMI was 27.97 ± 5.62 kg/m^2^. All subjects were men, with a mean hemoglobin level of 16.38 ± 1.34 g/dl at the time of donation. High hemoglobin levels are expected among blood donors and do not reflect the general population. The blood group distribution was as follows: 47.73% type O (n=179), 28.80% type A (n=108), 18.13% type B (n=68), and 5.34% type AB (n=20).

Measurement of anti-HCMV antibodies

**Figure 1:**
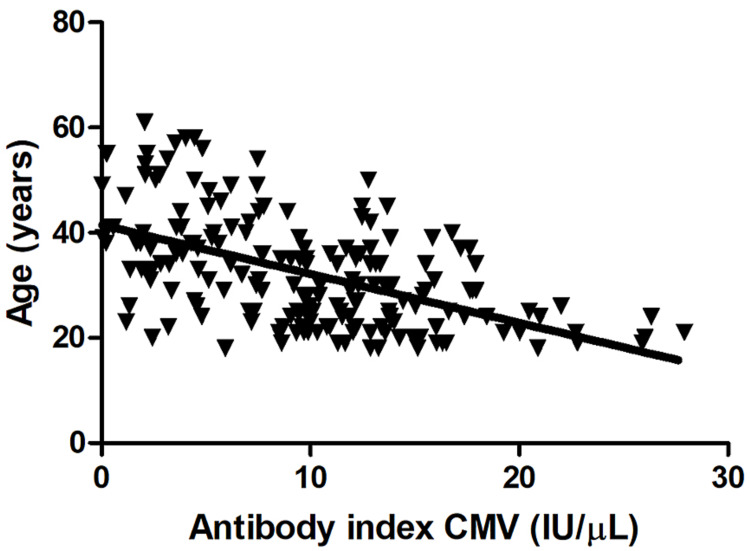
Distribution of anti-HCMV IgG levels according to different age groups Subjects were divided into three groups according to age: 18-30, 31-40, and 41-61 years. The 18-30-year group showed a significantly higher antibody level (median: 13.85, n=166, p<0.0001) than the 31-40-year group (median: 9.46, n=110). Additionally, the 31-40-year group showed a significantly higher antibody level (median: 9.46, n=110, p=0.0002) than the 41-61-year group (median 6.21, n=74). Mann-Whitney U test, two-tailed HCMV: human cytomegalovirus; IgG: immunoglobulin G

We performed ELISA to measure anti-HCMV IgG and IgM antibody levels among the blood donors. We found that the majority of donors were positive for anti-HCMV IgG antibody (95.73%, n=356), with a mean level of 10.98 ± 6.24 IU/ml. Anti-HCMV IgG levels were significantly different among the examined age groups. We stratified participants into three age groups: 18-30, 31-40, and 41-61 years. Participants in the 18-30-year group had significantly higher antibody levels (median: 13.85, n=166, p<0.0001) compared to those in the 31-40-year group (median: 9.46, n=110). Additionally, we found that those in the 31-40-year group showed significantly higher antibody levels (median: 9.46, n=110, p=0.0002) compared to those in the 41-61-year group (median: 6.21, n=74; Figure 1).

Only six subjects (1.56%) were positive for anti-HCMV IgM antibodies (1.56%), and all six of these subjects were also positive for IgG antibodies. No participants in the 18-30-year group were anti-CMV IgM antibody-positive or anti-CMV IgG antibody-negative. The 31-40-year group included four anti-CMV IgM antibody-positive and four anti-CMV IgG antibody-negative individuals, whereas the 41-61-year group contained two anti-CMV IgM antibody­-positive and 12 anti-CMV IgG antibody-negative individuals.

Association between anti-HCMV antibody levels and age

**Figure 2:**
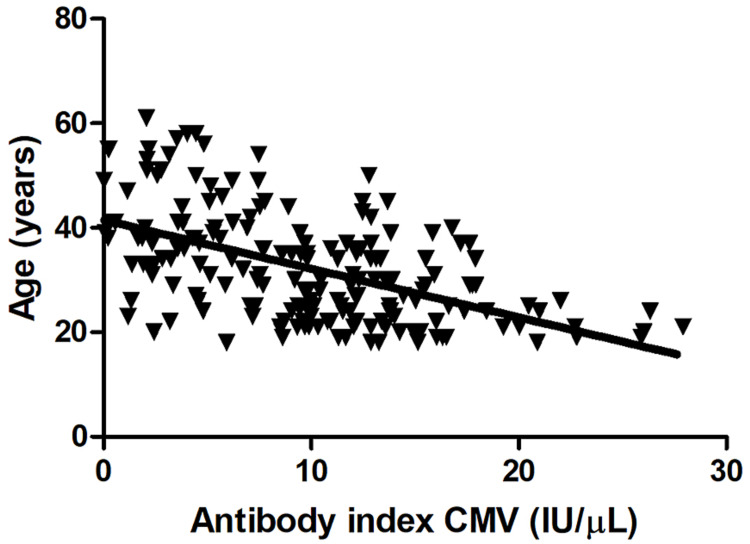
Correlation between anti-HCMV IgG levels and age A strong negative correlation between anti-HCMV IgG levels and age was identified (Pearson’s r=−0.51, 95% confidence interval: −0.59 to −0.43, F=125, p<0.0001) HCMV: human cytomegalovirus; IgG: immunoglobulin G

We examined the association between anti-HCMV antibody levels and participants’ age. We found a strong negative correlation between anti-HCMV IgG levels and age (Pearson’s r=−0.51, 95% confidence interval: −0.59 to −0.43, F=125, p<0.0001, Figure 2). In general, the level of anti-HCMV IgG antibodies decreased with increasing age.

Association of anti-HCMV antibody levels with BMI, hemoglobin levels, and blood groups

**Figure 3:**
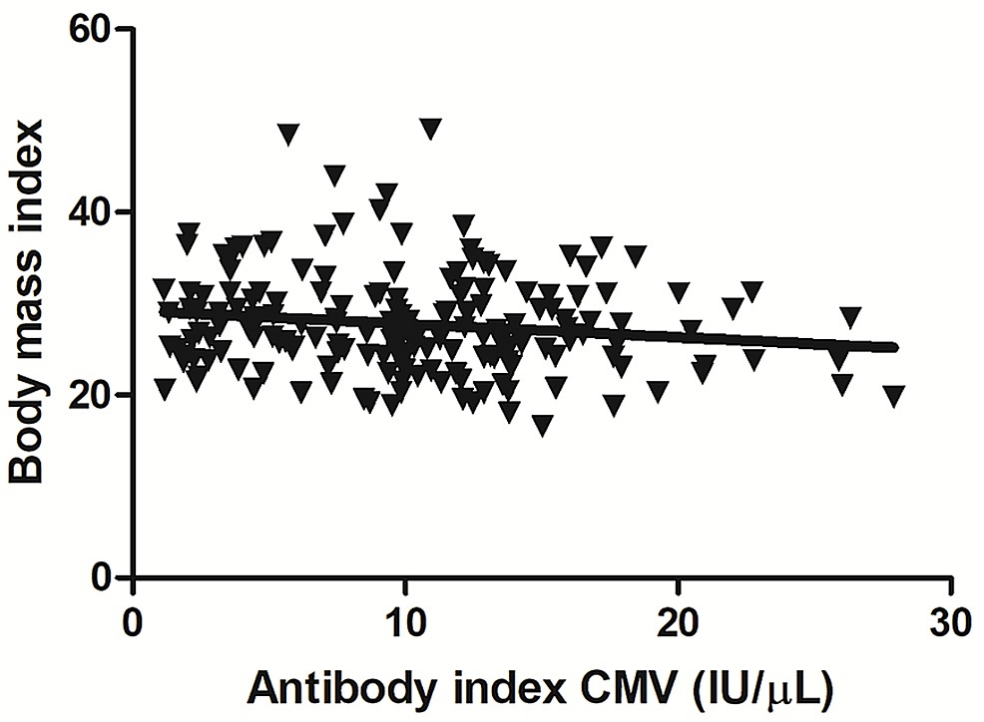
Correlation between anti-HCMV IgG levels and BMI A negative correlation was identified between anti-HCMV IgG levels and BMI (Pearson’s r=−0.11, p=0.036, F=4.42, 95% confidence interval: −0.21 to −0.007) BMI: body mass index; HCMV: human cytomegalovirus; IgG: immunoglobulin G

**Figure 4:**
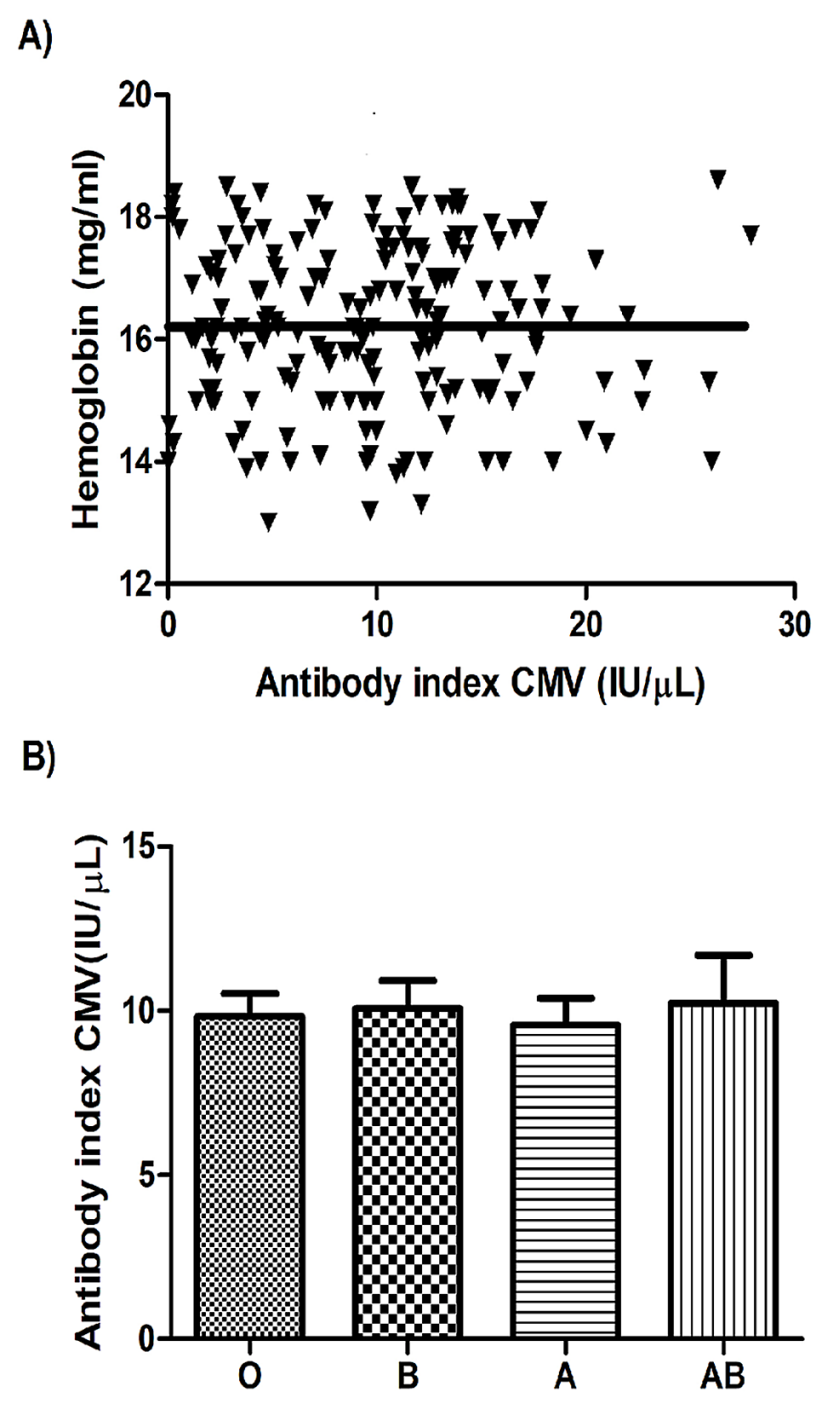
Correlation between anti-HCMV IgG levels and blood indices No significant correlation was found between anti-HCMV IgG levels and hemoglobin levels (A) or between anti-HCMV IgG levels and blood groups (B), p>0.05 HCMV: human cytomegalovirus; IgG: immunoglobulin G

To observe the relationships between antibody levels and other variables, we assessed the association between anti-HCMV antibody levels and participants’ BMI values. Figure 3 shows a significant decrease in anti-HCMV IgG antibody levels with increasing BMI (Pearson’s r=−0.11, p=0.036, F=4.42, 95% confidence interval: −0.21 to −0.007). No associations were found between anti-HCMV IgG levels and hemoglobin levels (Figure 4A) or between anti-HCMV IgG levels and blood groups (Figure 4B).

## Discussion

HCMV infection is a leading cause of congenital infections globally. In developed countries, HCMV infection is a common non-genetic cause of hearing loss in infants and a vital contributor to neurodevelopmental delay [19,20]. One study that was conducted on umbilical cord blood, which is used as a source of hematopoietic stem cells for transplantation, investigated the presence of viral DNA in donor blood samples and concluded that 2% of buffy coat samples (used as evidence of latent infection) were CMV-positive. Plasma sample testing was negative for CMV DNA, indicating no active infection. Moreover, none of all viral DNA-positive samples were found positive for CMV IgM antibody, but they were all positive for CMV IgG antibody by ELISA [21]. Another study showed that the CMV reactivation rate among patients receiving hematopoietic stem cell transplant is high, ranging from 30 to 70% [22].

In the current study, the seropositivity rate for IgG anti-HCMV antibody was 95.73% among male blood donors in Madinah, Saudi Arabia, indicating a high prevalence of previous viral exposure. Our results are in agreement with a national study, which showed a similar frequency among pregnant Saudi women (>96%) in the Asir region [23]. Another local study conducted in Jeddah, Saudi Arabia, reported a prevalence of HCMV infection of 80.7% among the studied population, but all participants in that study were non-Saudi individuals of various nationalities [24]. Another study performed in Jazan, Saudi Arabia, showed an HCMV IgG antibody positivity rate of 93.1% [25]. A study conducted in Brazil also showed a high prevalence of anti-HCMV IgG positivity (96.4%). Studies have reported high frequencies of anti-HCMV IgG positivity in Nigeria (74.2%), Bosnia (81.4%), and Sudan (84.56%) [26-28]. In contrast with our results and those of other studies, low frequencies of anti-HCMV IgG positivity were reported among blood donors in Germany (37.5%) and the UK (47.5%, in the "Understanding Society-the UK Household Longitudinal Study") [29,30].

Anti-HCMV IgM detection indicates a recent infection, and our study showed that only 1.6% of donors were IgM-positive. Our results fall within the range of 0.4-10% rate reported in other countries, such as Switzerland, Tanzania, and China [27-29,31]. Anti-HCMV IgM­ positivity indicates a recent infection (primary, reactivation, or reinfection), and anti-HCMV IgG has been detected in secondary (reactivation) HCMV infections in some HCMV-infected individuals [32]. The detection of anti-HCMV IgM alone cannot be used to diagnose primary infections because IgM antibodies can also be detected in secondary infections [33].

The presence of IgM-positive donors indicates a risk of transmission through blood transfusions to susceptible populations, such as immunosuppressed recipients, which is known as a transfusion-transmitted infection (TTI) [34]. Therefore, for this category of blood recipients, we recommend additional screening of donor blood for the presence of HCMV using a molecular method, such as reverse transcriptase-polymerase chain reaction (RT-PCR) or nucleic acid test (NAT).

In the current study, anti-HCMV IgG levels were negatively correlated with age. Donors in the 18-30-year group were all anti-HCMV IgG antibody-positive. The 31-40-year group included four donors who were anti-HCMV IgG antibody-negative, and the 41-61-year group included 12 seronegative donors, demonstrating the decrease in antibody levels with increasing age. This pattern is likely due to the waning of antibody levels with increasing age, which might be due to the reduced ability of memory B cells to yield high quantities or long-lasting plasma cells capable of producing antibodies [35]. Our findings are in agreement with previous studies that showed changes in anti-HCMV IgG levels with age [36,37]. Other study results align with that of ours, showing an inverse correlation between age and anti-HCMV IgG levels among pregnant women in KSA and China [23,38].

We found an inverse correlation between anti-HCMV IgG levels and BMI. However, our BMI-related results contrast with those of a study conducted among women, which showed no association between anti-HCMV IgG antibody levels and BMI [30]. This discrepancy could be due to the participation in the current study being limited to men. The impact of sex on the relationship between BMI and HCMV IgG levels requires further investigation, as immune responses are well-known to vary between men and women [39-41].

The present study showed no association between blood groups and anti-HCMV IgG levels. This finding is consistent with a study conducted in New Zealand that showed no association between blood groups and the response to HCMV infection [42]. However, a recent study on severe acute respiratory syndrome coronavirus 2 (SARS‑CoV‑2) showed that there was a correlation between antibody levels and blood groups [43].

Several methods are available for the prevention of HCMV infection via blood transfusions, such as leukodepletion [44], which could decrease the risk of leukocyte-associated adverse diseases, the incidence of human leukocyte antigen alloimmunization, and the transmission of viruses, such as HCMV, human immunodeficiency virus (HIV), and hepatitis viruses, during blood transfusion procedures [45,46]. Another option is limiting donation to HCMV-negative donors (as assessed both serologically and molecularly) [47,48].

The limitations of the current study include the relatively small sample size and the exclusion of female participants. Unfortunately, the rate of blood donation is low among women in KSA. Large, geographically varied, nationwide studies that include both genders are recommended for future investigations.

## Conclusions

Our study showed a high frequency of anti-HCMV IgG levels among healthy Saudi male blood donors. Therefore, blood banks should consider the application of preventative measures, such as leukodepletion or routine screening for HCMV and other potentially TTI-associated disease vectors, to prevent transmission to seronegative recipients. The study findings require further validation through large, comprehensive national studies. The prevention of disease transmission to transfusion recipients will reduce the burden associated with TTI-related complications. Implementing nationwide strategies to reduce viral transmission will be beneficial to healthcare providers worldwide.
